# A Case of Myosin Heavy Chain 9-Related Disorder Following Splenectomy Due to Misdiagnosis of Idiopathic Thrombocytopenic Purpura

**DOI:** 10.7759/cureus.55064

**Published:** 2024-02-27

**Authors:** Eren Arslan Davulcu, Emin Karaca, Nur Akad Soyer

**Affiliations:** 1 Hematology, University of Health Sciences, Bakirkoy Dr. Sadi Konuk Training and Research Hospital, Istanbul, TUR; 2 Medical Genetics, Ege University Faculty of Medicine, Izmir, TUR; 3 Hematology, Ege University, Izmir, TUR

**Keywords:** myh9-rd, eltrombopag, idiopathic thrombocytopenic purpura, inherited thrombocytopenias, myosin heavy chain 9-related disorder

## Abstract

This case study reports a patient with Myosin Heavy Chain 9 (MYH9)-related disorder (MYH9-RD) which is characterized by congenital macrothrombocytopenia, Döhle-like bodies, sensorineural hearing loss, cataracts, and glomerulopathy. Often misdiagnosed as idiopathic thrombocytopenic purpura (ITP), MYH9-RD requires accurate identification to avoid inappropriate treatments like steroids, rituximab, or splenectomy. Platelet transfusions were traditionally the only therapeutic option, but thrombopoietin receptor agonists (TPO-RA), specifically eltrombopag, have shown success in MYH9-RD treatment.

The case report involves a 27-year-old male with chronic ITP post-splenectomy, revealing thrombocytopenia, mild anemia, giant platelets, kidney failure, and hearing loss. Genetic testing identified a c.287C>T; p.(Ser96Leu) variant associated with MYH9-RD. Eltrombopag treatment, initiated before the definitive diagnosis, exhibited clinical and laboratory success. The study discusses the evolving landscape of treatments for inherited thrombocytopenias, emphasizing eltrombopag's efficacy, especially post-splenectomy, and its potential application in short-term preparations for elective surgeries.

The study underscores the importance of timely MYH9-RD diagnosis, preventing misdiagnoses and inappropriate treatments. Eltrombopag stands out as a potential therapeutic option, offering effective platelet count management, especially post-splenectomy, with ongoing research exploring alternative TPO-RAs. As MYH9-RDs are rare, increased awareness among healthcare professionals is crucial to ensure accurate diagnoses and optimal patient care.

## Introduction

Myosin heavy chain 9 (MYH9)-related disorders (MYH9-RD) belong to the group of inherited thrombocytopenias. It is caused by a mutation of the MYH9 gene, which encodes non-muscle myosin heavy chain-IIA (NMMHC-IIA) [1,2]. NMMHC-IIA belongs to the myosin superfamily of motor proteins. It is widely expressed across various hemopoietic cell types, where it serves crucial functions in multiple cellular processes. The protein is particularly involved in cell motility, cytokinesis, chemotaxis, phagocytosis, and the maintenance of cell shape [3,4].

MYH9-RD comprised five diseases with different clinical features: May-Hegglin anomaly, Sebastian syndrome, Fechtner syndrome, Epstein syndrome, and autosomal dominant deafness DFNA17 [5]. Although the predominant clinical picture changes in each disease, MYH9-RD is characterized by congenital macrothrombocytopenia, Döhle-like bodies in granulocytes, sensorineural hearing loss, cataracts, and glomerulopathy. The familial background could align with autosomal dominant inheritance, characterized by the presence of affected individuals, both males and females, across multiple generations. Alternatively, the affected person might be an isolated case within the family, termed a simplex case. Consequently, the lack of a documented family history should not exclude the possibility of making a diagnosis in such instances [5]. Diagnosis is established by clinical and laboratory findings supported by molecular genetic testing. In most cases, other clinical and laboratory findings are overlooked and MYH9-RD is misdiagnosed as idiopathic thrombocytopenic purpura (ITP).

While platelet transfusions were the only therapeutic option, thrombopoietin receptor agonists (TPO-RA) have been successfully used in MYH-9 RD. Suspecting MHY9-RD and initiating diagnostic genetic tests will prevent patients from misdiagnosing ITP and unnecessary treatments such as steroids, rituximab, and splenectomy.

## Case presentation

A 27-year-old male patient was consulted postoperatively because of chronic ITP after splenectomy for further follow-up. As far as it was learned from his history, he had been followed up in another center with a diagnosis of ITP since the age of three, and he received steroids, intravenous immune globulin several times, and platelet suspension when needed. While investigating because of proteinuria, he was diagnosed with chronic kidney failure four years ago and he has been prepared for renal replacement therapy. Although he did not have any complaints about hearing, mild hearing loss in one ear was also detected. His eye examination was normal. It was learned that there were individuals with thrombocytopenia in his family, but they were never examined in this respect.

**Figure 1:**
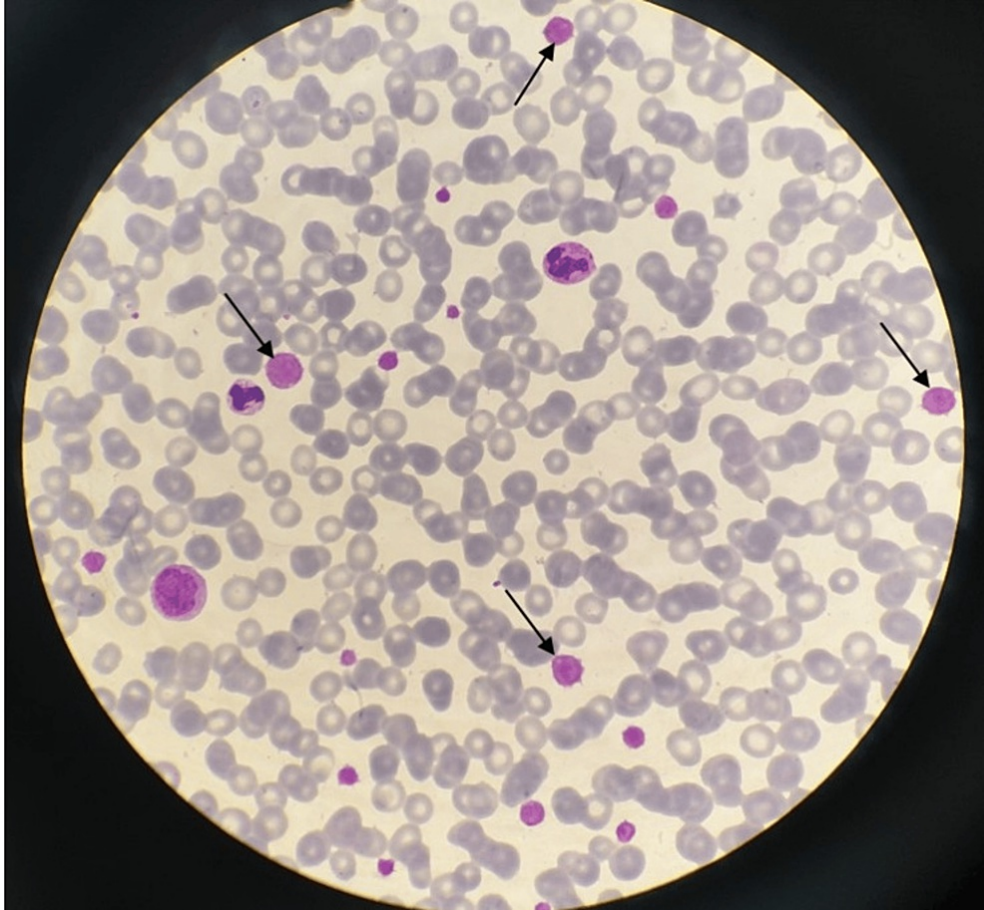
Peripheral blood smear demonstrating macrothrombocytes (black arrows)

In our initial hematologic examination one month after splenectomy, he had thrombocytopenia (4,000/mm^3^) and mild anemia (hemoglobin 9.52 g/dL). Giant platelets almost as big as an erythrocyte observed in peripheral blood smear (PBS) were very remarkable and the platelet count in PBS was calculated as 40,000/mm^3^. The Döhle body could not be observed in the granulocytes (Figure 1).

The coexistence of giant platelets, kidney failure, and hearing loss brought to mind the possibility of MYH9-RD. This way, genetic testing was performed considering that it might be MYH9-RD.

Meanwhile, renal replacement therapy was initiated and he had a complaint of bleeding from the catheter during dialysis. While waiting for the results of genetic testing, treatment with eltrombopag has been started. The drug dose was started at 50 mg/day and increased to 75 mg. Since the automatic blood counter could not be able to count giant platelets due to their size, there was no significant change in the peripheral blood count result but, platelet values evaluated by microscope up to 100,000/mm^3^ were observed in the PBS. Laboratory findings before and after eltrombopag treatment are summarized in Table 1.

**Table 1:** Laboratory findings before and after eltrombopag treatment WBC: White blood cell; HB: Hemoglobin; PLT: Platelet; PBS: Peripheral blood smear; MPV: Mean platelet volume

Parameters	WBC (/mm^3^)	HB (g/dL)	PLT (/mm^3^)	PLT (in PBS)	MPV	Creatinine (mg/dL)
Pre-eltrombopag	20,000	12	4,000	40,000	High	6
Post-eltrombopag	21,300	11.8	50,000	>100,000	High	5.3
Reference range	4,500-10,000	11.7-16	150,000-400,000	150,000-400,000	7.8-11	0.7-1.3

**Figure 2:**
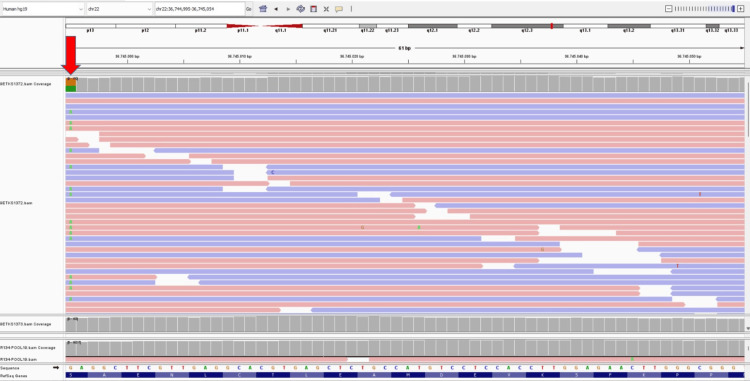
The mutation of the patient (c.287C>T (p.Ser96Leu)) in the MYH9 gene (red arrow). An integrative genomics viewer (IGV) screenshot showing the sequencing reads (grey bars) mapping to exon 8 of the MYH9 gene located at genomic position g.36,744,995 on chromosome 22. The reference nucleotide sequence and the amino acid translation are provided under the sequencing reads. The heterozygous substitution of a cytosine (C) to a thymine (T) at nucleotide position of 287 MYH9: Myosin Heavy Chain 9

The patient was found to have a c.287C>T; p.(Ser96Leu) mutation in polymerase chain reaction amplification of the targeted genomic region which was sequenced on a next-generation sequencing platform (Figure 2).

This mutation in the MYH9 gene is associated with MYH9-RD [6]. Considering the clinical and laboratory findings of MYH9-RD diseases, the presence of macrothrombocytopenia, nephritis and hearing loss, absence of cataracts, and inclusion bodies reveal the diagnosis of Epstein syndrome in our patient [7]. Eltrombopag treatment, initiated before the diagnosis of MYH9-RD, has been continued at 50 mg/day because of clinical and laboratory successful and satisfactory responses. Current literature data also support using eltrombopag in MYH9-RD [8,9]. In addition, a genetic consultation was planned for the patient's family.

## Discussion

While platelet transfusions are the only treatment option for inherited thrombocytopenias, new treatment options are emerging as the pathogenesis of the disease is understood. Defective megakaryocyte differentiation and maturation, defective proplatelet formation, and reduced platelet lifespan were involved in the pathogenesis of inherited thrombocytopenias [10,11]. The opinion that eltrombopag, a TPO agonist, would be beneficial in inherited thrombocytopenias came from this information and has been successful in MYH9-RD without major side effects [9,12]. Furthermore, the study from Pecci et al. revealed that the only baseline characteristic predicting treatment response to eltrombopag was the previous splenectomy. All the asplenic patients achieved major responses (platelet count higher than 100,000/mm^3^) and the mean platelet counts of splenectomized patients were statistically higher than non-splenectomized patients (151,000/mm^3^ vs 81,000/mm^3^, p=0.04) [9]. In MYH9-RD, eltrombopag doses up to 75 mg/day have been reported [9,12,13].

Eltrombopag is also useful in short-term use to prepare patients with MYH9-RD and severe thrombocytopenia for elective surgery, thus avoiding platelet transfusions. It is advised to initiate eltrombopag three weeks before the procedure, up to 75 mg/day, until 3-7 days after surgery [13]. It has been reported that using eltrombopag, chemotherapy was safely administered to a patient with pancreatic cancer diagnosed with MYH9-RD [14] and a cesarean section was successfully performed on a pregnant woman with MYH9-RD [15].

Since side effects such as cataracts and increased bone marrow fibrosis have been reported in the long-term use of eltrombopag for other diseases, caution should be exercised in this regard [16].

The successful use of romiplostim, another TPO-RA in MYH9-RD has been reported in a mouse study [17] and a case report of a patient with Fechtner syndrome [18]. Recent data demonstrated the first use of avatrombopag, a novel TPO-RA, following failed treatment with eltrombopag in a patient with MYH9-RD [19].

## Conclusions

Since MYH9-RDs are rare diseases, their diagnoses can be missed and different treatments can be applied due to potential diagnosis of other thrombocytopenic disorders as in our patient who had splenectomy with a diagnosis of ITP. For hereditary thrombocytopenias such as MYH9-RD, where platelet transfusion is the main treatment, eltrombopag may have a potential role as an effective therapeutic option. Cases of successful use of other TPO agonists have been reported.
